# Report of Cholangiocarcinoma With Transheterozygous BRCA1 and BRCA2 Co-mutation

**DOI:** 10.7759/cureus.60767

**Published:** 2024-05-21

**Authors:** Nicholas Prabhakar, Harrah Chiang, Edward Nabrinsky, John Eklund

**Affiliations:** 1 Internal Medicine, Hematology and Oncology, Advocate Lutheran General Hospital, Park Ridge, USA; 2 Internal Medicine, Advocate Lutheran General Hospital, Park Ridge, USA; 3 Hematology and Oncology, Advocate Lutheran General Hospital, Park Ridge, USA

**Keywords:** biliary, genetics, transheterozygous, brca, cholangiocarcinoma

## Abstract

Cholangiocarcinoma is an aggressive malignancy involving the epithelial cells of the intrahepatic, perihilar, or extrahepatic biliary tree. It is a disease that is often diagnosed late in its course and progresses quickly. Identifying genomic mutations may provide an important utility in predicting disease course and individualizing therapy for these patients. Mutations in BRCA1 or BCRCA2 genes have been increasingly documented in hepatobiliary malignancies, but they remain a relatively uncommon occurrence. Co-mutations in both BRCA1 and BRCA2 genes are even rarer, with no previously documented reports to our knowledge of BRCA co-positivity in a patient with a hepatobiliary malignancy. We present a case of a patient with cholangiocarcinoma found to have mutations in both BRCA1 and BRCA2 genes.

## Introduction

Cholangiocarcinoma is a malignancy involving the epithelial cells of the bile duct, which includes the intrahepatic, perihilar, or extrahepatic biliary tree [1]. It is a rare malignancy that is typically advanced by the time of discovery. It is the second most common primary hepatic malignancy, with incidence rates ranging from 0.8 to 2 per 100,000, and accounts for 3% of malignant gastrointestinal tumors [2]. They consist of 90-95% adenocarcinomas and 5-10% squamous cell carcinomas. It typically has an aggressive disease course and is often difficult to treat effectively with existing methods. This is compounded by the fact that there is currently no effective screening method [3]. Given this, there has been increased attention recently regarding genomic profiling of these patients to better predict disease course and individualize treatment for patients that fail first-line treatment. There are multiple documented genomic mutations associated with cholangiocarcinoma [4,5], and this includes the BRCA1 and BRCA2 genes. BRCA1 and BRCA2 are tumor suppressor genes with mutation prevalence in 0.2-0.3% of the general population, although this varies with various populations, most notably with a higher rate of occurrence in the Ashkenazi Jewish population [6]. Mutations in BRCA1 or BRCA2 can lead to an increased risk of malignancy when there is a loss of heterozygosity, leading to a deficiency of the protective mechanisms they normally encode, which thereby leads to genomic instability and a higher potential for transformation to malignancy [7,8]. Although BRCA-associated cholangiocarcinomas are uncommon, even rarer is a mutation in both BRCA1 and BRCA2 genes. Co-mutation of BRCA1 and BRCA2 genes, also known as transheterozygosity (TH), is rare in the general population. There are only a few other documented instances of malignancies expressing double-BRCA mutations, seen with single case reports or case series in colorectal, gastric, lung, lymphoma, pancreatic, and prostate carcinomas. We present the first reported case of a patient diagnosed with cholangiocarcinoma found to have mutations in both BRCA1 and BRCA2 genes. 

## Case presentation

**Figure 1:**
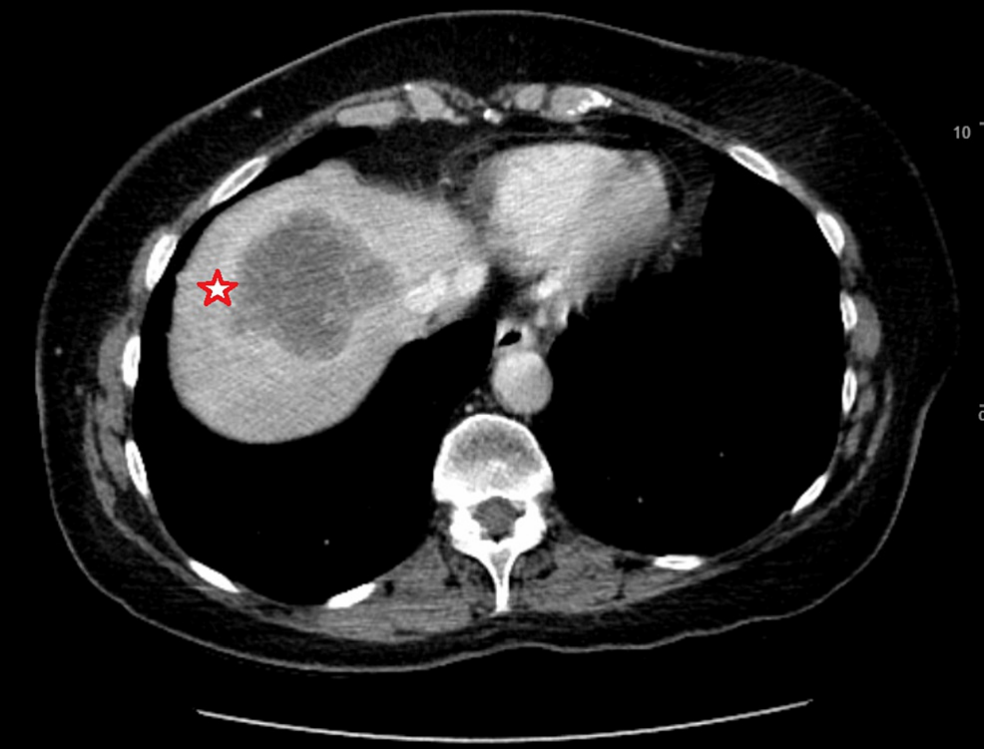
Large hypodense mass in the dome of the liver (star in image).

The patient is a 71-year-old female who developed a right upper quadrant and weight loss in November 2019. She subsequently developed hematuria, for which a CT urogram ordered in spring 2020 demonstrated a large hypodense mass in the dome of the liver with perilesional enhancement, in addition to an irregular soft tissue mass in the right upper quadrant mesentery (Figure 1). She underwent a fine needle aspiration of the liver mass that was positive for adenocarcinoma with necrosis, favoring an upper gastrointestinal tract or pancreaticobiliary primary tumor. PET CT scan demonstrated a large lobular, necrotic-appearing hypermetabolic lesion in the dome of the right hepatic lobe measuring 6.0 cm with an standardized uptake value (SUV) of 9.7, as well as an additional tubular-appearing hypermetabolic lesion in the right upper quadrant adjacent to the small bowel and extrahepatic, with an SUV of 7.9. 

The patient underwent FoundationOne genetic testing from peripheral blood and was found to have BRCA1 L639fs*51 as well as BRCA2 T3033fs*11 somatic mutations. Germline testing revealed a pathogenic BRCA2 T3033fs*11 mutation. The patient was diagnosed with cholangiocarcinoma and started treatment with intravenous cisplatin and gemcitabine. A CT scan after five cycles of therapy showed decreasing size in the necrotic-appearing mass in the dome of the right heparin lobe, for which she underwent exploratory laparoscopy with open segment 8 liver resection six months after diagnosis; pathology demonstrated necrosis, fibrosis, and chronic inflammation and was negative for malignancy, suggestive of complete response to chemotherapy. 

CT scan obtained three months after surgery demonstrated focal enhancement at the dome of the liver with postoperative changes for which the patient underwent a biopsy that demonstrated recurrent adenocarcinoma of pancreaticobiliary primary. She resumed chemotherapy. Six months later, the patient progressed with increasing disease in the liver on PET CT scan, for which she was switched to pembrolizumab immunotherapy monotherapy for 10 months prior to progressive disease to her lungs. 

The patient subsequently started olaparib 300 mg twice daily monotherapy, which she has continued for the previous 18 months. The most recent PET CT, 15 months into therapy, demonstrated one site of isolated metastasis in the liver dome without evidence of other metastatic disease. She continues to tolerate therapy well. 

## Discussion

As mentioned previously, cholangiocarcinoma is an aggressive malignancy of the intrahepatic, perihilar, or extrahepatic biliary tree [1]. There are multiple associated predisposing risk factors, including primary hepatobiliary diseases such as primary sclerosing cholangitis (PSC), cholelithiasis, cholecystitis, chronic liver disease, genetic disorders, carcinogens, specifically Thorotrast and nitrosamines, and liver fluke infections, including clonorchiasis and opisthorchiasis [3]. Another aspect that has received further examination recently is the genomic profiles of these patients. Information on genomic mutations in cholangiocarcinoma has steadily increased in recent years with the rise of more available gene sequencing methods, and this has increasingly shown promise in its usefulness. Given the aggressive nature of an often treatment-resistant disease, combined with a lack of effective screening methods, identification of genomic mutations can prove useful both with patient prognostics and individualizing treatment. Commonly associated genomic mutations associated with cholangiocarcinoma include KRAS, BRAF, p52, and SMAD4 genes [4,5]. Prevalence and disease course for patients with these mutations tend to vary based on whether the disease is an intrahepatic or extrahepatic cholangiocarcinoma. Alterations can also differ depending on gender [9]. BRCA1 and BRCA2 gene mutations have also attracted increased interest in regard to patients with hepatobiliary malignancies. 

Over the last decade, researchers have thoroughly investigated the tumor suppressor genes BRCA1 and BRCA2. The mechanism that leads to malignancy occurs when there is a loss of heterozygosity, namely a loss of functioning mutation with the normal allele, leading to an abnormally functioning protein product. The proteins that these genes encode are expressed in many tissues in the body in a cell-cycle-dependent manner, with the highest function in cell replication. BRCA1 and BRCA2 gene products normally work to preserve cell cycle and chromosomal structure both with the cellular response to DNA damage as well as DNA double-strand break repair via homologous recombination [7,8]. The deficiency of these mechanisms therefore leads to genomic instability and a higher potential for transformation into malignancy. While it is most commonly associated with breast and ovarian cancers, it has been increasingly found to be present in biliary cancers. Overall occurrence of BRCA mutations in biliary tract cancers has been reported as 3.6% with a BRCA2 predominance (0.6% have a BRCA1 mutation and 3% have a BRCA2 mutation) [10]. Early data showed an increased relative risk of 4.97 (95% CI 1.50-16.52) for BRCA2 carriers for developing gall bladder or bile duct cancer [11]. Although BRCA-associated cholangiocarcinomas are uncommon, even rarer is a mutation in both BRCA1 and BRCA2 genes. 

BRCA1 and BRCA2 genes transheterozygosity is rare in the general population but is found to have a similar probability of developing breast cancer, ovarian cancer, or both cancers as compared to single mutations [12]. In the few patients found with BRCA TH, patients are diagnosed at a younger age than in patients with single mutations. The mean age of breast cancer diagnosis in this population is 40.8 years old, while with ovarian cancer it is 45.7 years old [13]. Based on a literature search, there are only a few other malignancies that have shown expression of double-BRCA mutations and are seen with single case reports or case series in colorectal, gastric, lung, lymphoma, pancreatic, and prostate carcinomas [14,15]. In BRCA1/2 mutations, there are co-mutations in high frequency with other genes that are involved in DNA repair, which include KRAS, MET, and MDM2/6 [9]. However, to our knowledge, this is the first reported case of cholangiocarcinoma with mutations in both BRCA1 and BRCA2. The patient was treated with olaparib, a poly-adenosine diphosphate-ribose polymerase (PARP) inhibitor, that is typically used as a maintenance treatment in germline or somatic BRCA gene cancers, commonly ovarian cancer [16]. The discovery of transheterozygosity of the BRCA 1 and 2 genes in her cholangiocarcinoma composition allowed for the use of targeted therapy such as olaparib. This BRCA1/2-targeted approved therapy, in addition to chemotherapy and surgical resection, has thus far yielded positive results in our patient. Further study of this case, as well as future transheterozygous BRCA1 and BRCA2 cholangiocarcinomas, may provide further information on patients of this population and define a more targeted approach for individualized treatments. 

## Conclusions

We describe a case of a woman with cholangiocarcinoma with transheterozygous BRCA1 and BRCA2 co-mutations who failed initial chemotherapy and was treated with targeted therapy for BRCA-positive patients. This case is unique in that it is the first report to our knowledge of BRCA1 and BRCA2 co-positivity in a patient with a hepatobiliary malignancy. Further studies will prove useful in examining the disease course and treatment outcomes of patients with this transheterozygosity. 
